# War

**Published:** 1914-09

**Authors:** 


1854-56 Crimean War; 1857 Sepoy Mutiny; 1859 Italy and France vs. Austria; 1860-65 Civil War in United States; 1861-67 Austria and Allies vs. Mexico; 1864 Prussia and Austria vs. Denmark; 1866 Austria vs. Prussia; 1870 Franco-Prussian War; 1877-78 Russo-Turkish War; 1885 Servia vs. Bulgaria; 1894-95 Japan vs. China; 1897 Greece vs. Turkey; 1898 Spanish-American War; 1899 Filipino-American War; 1900 China vs. Allies; 1899-'02 Boer War; 1904 Russia vs. Japanese; 1912 Balkan vs. Turkey; 1913 Balkan vs. Bulgaria; 1914 Mexican War.

None of these wars has involved so complicated a strife as has now begun nor can the present great European conflict be accurately compared with the frank attempts at conquest of Napoleon, of Caesar, whose name has persisted as a title for two of the monarchs engaged at present, to other ancient wars of conquest nor to the crusades. While offensive and defensive alliances of independent nations is no new thing in warfare, so general an involvement of countries having no immediate desire or excuse for war and such peculiar problems involving neutral nations, have not hitherto been witnessed.

As in the case of disease, the utter insignificance of the exciting cause as compared with the predisposing cause, is well illustrated in the present war. To the best of our comprehension, the theoretic cause of the war is the endeavor to maintain the balance of power in Europe; the actual cause, the hope of overthrowing this balance in the interests of one or another nation. For many years, Europe has been like a piece of badly annealed glass, ready to explode at any jar or rise of temperature. The rapidity with which the war has proceeded well supports this view. There have been no General McClellans in Europe to arouse the impatience of an army, a people or an interested bystander. And, in justice to the memory of General McClellan, it must be recognized that there has been a state of preparedness, in all military ways, in Europe, entirely unlike the condition with which the latter had to contend. In a number of instances, during the Civil War, the contestants repeated their manoeuvres upon the same ground; in other instances, they fought over ground upon which Revolutionary battles had been waged. In Europe, almost every strategic point is a veritable Armageddon, upon which wars have been fought for centuries, sometimes since prehistoric times, and familiar to many of the foreign contestants, even in the ranks, on account of the short distances, historic interest and facilities for travel, thus affording a marked contrast to conditions prevailing during the Civil War.

Modern inventions have rapidly changed the conduct of warfare, even in very recent years. Aerial conflicts, predicted in yellow literature, wireless communication, improved destructive agencies, in connexion with the familiarity with even minute geographic and strategic details and the state of preparedness already mentioned, duplicate in rapidity of action and sensationalism, the wildest flights of imagination of the author of a cinematograph play.

Certain of the developments of applied science and of social economics, bring up live issues of international law. For centuries, the high seas have been established as beginning three miles from land. Wireless communication has already withdrawn some of the most cogent reasons for placing the high seas under a law of their own. The efficiency of modern guns will inevitably produce a clash between the theory of the high seas and both the theory and the practical requirements of neutrality. Similarly, the rapidity with which news is transmitted and with which actual hostilities may be begun, bring up embarrassing questions in regard to the right of a nation to seize merchant craft of adversaries. Persons and property of neutral nations, embarked upon vessels in time of peace, have already been seized or, at least pursued. Enormous financial loss, personal inconvenience and danger have been brought about on account of the celerity of modern warfare. Both for military purposes and the ordinary needs of the non-combatant population, as well as out of respect for the rights of neutral nations, it would seem that some readjustment of international law must follow this war, to provide that a journey or transportation begun in time of peace may be completed without jeopardy, that naval fights shall not endanger occupants of a peaceful shore, and that the ordinary world-wide commerce of the neutral portion of the world shall not be interfered with, except so far as the trade with warring nations is concerned—and perhaps not even then, since the needs of the contestants are actual and pressing. We need not go out of our own homes in this neutral nation, nor beyond the scope of our domestic necessities, to realize how directly this war on the other side of the world damages our own financial condition·

To a large degree, the state of tension in Europe, especially in regard to internecine dangers and weaknesses of nations, is due to the compulsory political union of different and even discordant races or, at least, linguistic groups. For example, the disintegration of Poland which, at the beginning of the sixteenth century was regarded as the largest, strongest and most cultured nation of Europe, and the union of part of the German with part of the Slavic races, the Gallicizing of part of the Low German portion of the western coast, the establishment of neutral states by international treaties, the defensive alliance of nations in groups that appear quite arbitrary, have brought about the most peculiar problems within a few days of the beginning of hostilities. Yet, in Alsace-Lorraine, the allegiance of many to their conquerors of only a few generations ago rather than to their recent conquerors of the same race, is noteworthy while, in this country, recent foreign ancestry or even foreign birth does not cause the general racial partisanship which might be expected.

So far as our own country is concerned, provided that it is not engaged in the general hostilities, we feel much more optimistic than most persons. By the withdrawal of reservists—although practical obstacles appear to be reducing this factor to a minimum—by the enforced restriction of immigration during the war, and by the demand and room for men which such a war is bound to leave behind for many years, time will be given for the assimilation of foreign blood and ideas. Without race prejudice of any kind, such assimilation is generally recognized to be needful for any nation. Indeed, in our experience it is most frankly stated and with the maximum of race prejudice by those who are themselves very recently of foreign extraction.

Following the initial rise of the price of various commodities and the check on commerce and business which interference with free international intercourse renders inevitable, we can see only two alternatives: either this country will have an abundance of natural and manufactured necessities of life for its own consumption, with a resultant decrease of prices, or else there will be so great a demand for all sorts of products by nations whose own production is hampered by war while their demands are increased, that, in spite of higher prices we shall be in the state of a prosperous merchant whose store is besieged by a crowd of purchasers.

In strictly medical ways, the war interests the medical profession. Many of its members have been subjected to inconvenience, even to hardship and danger by its sudden advent. International medical meetings announced, have been indefinitely postponed. Foreign study by American medical men will be greatly interfered with during the war. For instance, we have just destroyed an announcement of courses in Paris which we expected to publish in this issue, since, if carried out, they would be practically inaccessible. Domestic post-graduate study must be developed to meet the corresponding demand, at least temporarily, and we anticipate that a very marked permanent change of view in this particular, will result. With some reluctance, it must be admitted that military sanitation and hygiene, if not military surgery and administration as carried out during the present war, will probably be based on object lessons derived from the Japanese-Russian

War, rather than from that of the Spanish-American or British-Boer War. In due time, if possible, we hope to secure through our European editorial staff, articles dealing with military medicine. At present, difficulties of communication, not to mention the prematureness of such articles until sufficient time for actual experience has elapsed, render it impossible to make definite promises of this nature.

